# Use of a beta-lactam graded challenge process for inpatients with self-reported penicillin allergies at an academic medical center

**DOI:** 10.3389/falgy.2023.1161683

**Published:** 2023-07-31

**Authors:** Shawnalyn W. Sunagawa, Scott J. Bergman, Emily Kreikemeier, Andrew B. Watkins, Bryan T. Alexander, Molly M. Miller, Danny Schroeder, Erica J. Stohs, Trevor C. Van Schooneveld, Sara M. May

**Affiliations:** ^1^ Department of Pharmaceutical and Nutrition Care, Nebraska Medicine, Omaha, NE, United States; ^2^ Department of Internal Medicine, University of Nebraska Medical Center, Omaha, NE, United States; ^3^ Department of Pharmacy, St. Dominic Jackson Memorial Hospital, Jackson, MS, United States

**Keywords:** penicillin allergy, graded challenge, graded challenge (test dose), penicillin allergy delabelling, penicillin allergy screening algorithm

## Abstract

**Background:**

The Antimicrobial Stewardship Program (ASP) at Nebraska Medicine collaborated with a board-certified allergist to develop a penicillin allergy guidance document for treating inpatients with self-reported allergy. This guidance contains an algorithm for evaluating and safely challenging penicillin-allergic patients with beta-lactams without inpatient allergy consults being available.

**Methods:**

Following multi-disciplinary review, an order set for beta-lactam graded challenges (GC) was implemented in 2018. This contains recommended monitoring and detailed medication orders to challenge patients with various beta-lactam agents. Inpatient orders for GC from 3/2018–6/2022 were retrospectively reviewed to evaluate ordering characteristics, outcomes of the challenge, and whether documentation of the allergy history was updated. All beta-lactam challenges administered to inpatients were included, and descriptive statistics were performed.

**Results:**

Overall, 157 GC were administered; 13 with oral amoxicillin and 144 with intravenous (IV) beta-lactams. Ceftriaxone accounted for the most challenges (43%). All oral challenges were recommended by an Infectious Diseases consult service, as were a majority of IV challenges (60%). Less than one in five were administered in an ICU (19%). Almost all (*n* = 150, 96%) were tolerated without any adverse event. There was one reaction (1%) of hives and six (4%) involving a rash, none of which had persistent effects. Allergy information was updated in the electronic health record after 92% of the challenges.

**Conclusion:**

Both intravenous and oral beta-lactam graded challenges were implemented successfully in a hospital without a regular inpatient allergy consult service. They were well-tolerated, administered primarily in non-ICU settings, and were often ordered by non-specialist services. In patients with a self-reported penicillin allergy, these results demonstrate the utility and safety of a broadly adopted beta-lactam GC process.

## Introduction

Penicillin allergy is reported in approximately 7%–10% of patients, which makes it one of the most common medication allergies (1, 2). Recent literature shows that more than 90% of individuals who report penicillin allergy are found to not be allergic after appropriate evaluation (3). Inaccurate labeling of patients with a penicillin allergy can create barriers to appropriate antimicrobial management. For example, providers may be hesitant to utilize other beta-lactam agents in patients with a penicillin allergy due to concerns for cross-reactivity, as previous reports of general cross-reactivity rates were around 10%, despite more recent and rigorously conducted literature demonstrating rates as low as 1% in penicillin-allergic patients who are given a first-generation cephalosporin or agents with a similar R1 sidechain (4). The concern for cross-reactivity may lead to the selection of a drug that has a broader spectrum, is more expensive, associated with more adverse effects, or is non-preferred for the given infection (3).

Both patients and providers have been hesitant to administer cephalosporins and carbapenems to patients with severe penicillin allergies, even with low rates of reported cross-reactivity. Thus, in 2017, Nebraska Medicine's Antimicrobial Stewardship Program (ASP) in conjunction with the University of Nebraska Medical Center's Division of Allergy and Immunology created a Penicillin Allergy Guidance Document (5) with a corresponding Graded Challenge Order Set to address these general barriers to appropriate antimicrobial management encountered in patients with reported penicillin allergies. This guidance document contains descriptions of allergic reaction types, rates of cross-reactivity with different agents, and an algorithm for challenging penicillin allergic patients. In 2022, the Joint Task Force on Practice Parameters in conjunction with the American Academy of Allergy, Asthma, and Immunology and the American College of Allergy, Asthma, and Immunology (AAAAI/ACAAI) published a practice parameter update, which emphasized the preferential use of drug challenges as opposed to skin testing in patients with certain reported drug allergies (6). Specifically, the parameter update focused on single to multi-step graded challenges and a proactive approach to de-labeling patients with penicillin allergies.

The following report describes an updated analysis (7) on our use of a graded challenge process and the associated changes to the Penicillin Allergy Guidance Document as a result of the Practice Parameter update. The focus of this review was to assess characteristics of patients undergoing graded challenges, outcomes of the provocation, and subsequent allergy documentation of beta-lactam graded challenges in patients with a self-reported penicillin allergy.

## Methods

### Quality improvement project overview

Patients with orders for a graded challenge with oral amoxicillin (January 2019–June 2022) or intravenous beta-lactam (March 2018–June 2022) were retrospectively reviewed. Patients were included if they were admitted to Nebraska Medicine and received an intravenous or oral graded challenge and had a reported beta-lactam allergy documented. If patients received single doses of amoxicillin for indications other than an oral challenge, they were excluded. Patients were selected by providers to receive a graded challenge based on the need to receive a beta-lactam antibiotic and ASP's Penicillin Allergy Guidance document for treating inpatients with self-reported allergy.

### Graded challenge order set

Intravenously administered graded challenges were standardized through an order set within the institution's electronic health record (EHR). This order set contains various beta-lactam antibiotics with detailed administration instructions, rescue medications, associated monitoring to challenge penicillin allergic patients, and requires providers to acknowledge that the patient provided verbal consent, per our institution recommendation, to undergoing the graded challenge. This process was designed to be used for low-risk patients defined as having a non-IgE mediated allergy, or any reaction reported greater than 10 years ago, including IgE mediated reactions, except a severe cutaneous adverse reaction. The challenge was administered with test dose infusions of 1% of the target dose, then 10%, and finally the full dose, each 30 min apart. Oral amoxicillin was not included in the graded challenge order set for the time frame included; these challenges were constructed manually based on the safety parameters from the intravenously administered order set and doses were selected based on previously reported literature (8–21).

### Data definitions and outcomes

The EHR-abstracted data included characteristics of the graded challenge (i.e., antimicrobial agent, indication for graded challenge, patient level of care, and infectious diseases consultation) and patient characteristics (i.e., demographics and allergy history). Historical penicillin reactions were recorded and categorized based on the Penicillin Allergy Guidance Document.

This review aims to assess ordering, outcomes of the challenge, and allergy documentation. The primary outcome was safety of the graded challenge as defined by characterization of reaction to the graded challenge. Other outcomes included appropriateness of graded challenge, use of rescue medications, need for intensive care unit (ICU) care due to allergic reaction, days of therapy for challenged antibiotic class, and subsequent antibiotic used.

### Data collection and analysis

Data was abstracted from the EHR by manual review. Descriptive statistics were used including counts and percentages for categorical variables and means and medians for continuous data. This project was assessed by the Institutional Review Board at the University of Nebraska Medical Center as a quality improvement project and the need for full IRB review was waived.

## Results

### Graded challenges general use

Of the 161 intravenous graded challenges ordered, 147 were administered and 144 were IV beta-lactams. The others were discontinued prior to administration for reasons such as new culture susceptibility results. Additionally, of the 216 one-time amoxicillin orders, 203 were excluded due to indications other than an oral challenge. Thirteen oral amoxicillin challenges were administered. See Table 1 for a detailed description of patient characteristics.

**Table 1:** Patient and graded challenge characteristics.

Intravenous graded challenge (*N* = 144)^a^	
**Age (years), average (range)**	59.6 (6–91)
**Male sex**	59 (41%)
**Immunocompromised status**	
Immunocompetent	97 (67%)
Hematology-oncology/BMT	29 (20%)
Solid organ transplant	3 (2%)
HIV	3 (2%)
Other	12 (8%)
**Antibiotic allergies, average no. listed (range)**	2.4 (1–20)
**Reported penicillin allergy reaction (*n* = 124)** **^b^**	
Hives	40 (32%)
Anaphylaxis	26 (21%)
Unknown	25 (20%)
Rash	12 (10%)
Other, non-allergic side effect	21 (17%)
**Indication for IV GC**	
Empiric treatment	90 (63%)
Directed treatment	54 (37%)
**Infectious diseases (ID) consult**	93 (65%)
ID recommended IV challenge	86 (60%)
**Location IV GC administered**	
Inpatient ward	108 (75%)
Intensive care unit	28 (19%)
Emergency department	6 (4%)
Step-down unit	2 (1%)
**IV GC antibiotic**	
Ampicillin	4 (3%)
Ampicillin/sulbactam	4 (3%)
Aztreonam	1 (1%)
Cefazolin	7 (5%)
Cefepime	41 (28%)
Ceftazidime/avibactam	2 (1%)
Ceftolazone/tazobactam	1 (1%)
Ceftriaxone	62 (43%)
Ertapenem	6 (4%)
Meropenem	3 (2%)
Nafcillin	1 (1%)
Oxacillin	2 (1%)
Penicillin G	5 (3%)
Piperacillin/tazobactam	5 (3%)

Oral amoxicillin challenge (*N* = 13)^a^	
**Age (years), average (range)**	62.5 (35–76)
**Male sex**	7 (54%)
**Immunocompromised status**	
Immunocompetent	8 (62%)
Hematology-oncology/BMT	3 (23%)
HIV	1 (8%)
Solid organ transplant	1 (8%)
**Antibiotic allergies (no.), average (range)**	1.6 (1–3)
**Reported penicillin allergy reaction**	
Unknown	6 (46%)
Hives	3 (23%)
Rash	3 (23%)
Anaphylaxis	1 (8%)
Other, non-allergic side effect	0 (0%)
**Indication for PO challenge**	
Empiric treatment	10 (77%)
Directed treatment	3 (23%)
**ID consult**	13 (100%)
ID recommended PO challenge	13 (100%)
**Location administered**	
Inpatient Ward	13 (100%)
**Amoxicillin dose**	
250 mg × 1 dose	11 (85%)
Other^c^	2 (15%)

^a^ All values are reported as number (percent) unless otherwise noted.

^b^ 23 patients had other beta-lactam allergies listed, but did not have a specific reported penicillin allergy listed.

^c^ One patient was administered a 2-step oral challenge (25 mg × 1 dose followed by 250 mg × 1 dose) and one patient was administered a 125 mg × 1 dose.

For the intravenous graded challenges (Table 1), the most common antibiotics challenged were ceftriaxone (43%) and cefepime (29%). The majority of the intravenous graded challenges were administered in a non-ICU setting (80%) and were recommended by an Infectious Diseases (ID) service (60%). All oral amoxicillin challenges were administered in a non-ICU setting and all were recommended by an ID service. The most common oral amoxicillin challenge dose was a one-time 250 mg dose (85%).

### Graded challenge safety, appropriateness, and allergy updates

Of the 144 intravenous beta-lactam graded challenges, 137 (96%) were tolerated without any documented adverse event (Table 2). Of the seven with documented adverse events (Table 3), one developed hives that resolved after one dose of oral diphenhydramine. The other six adverse events were either rash or itching. One of these six patients did not require any rescue medications, while the other five patients experienced resolution of rash or itching after one dose of oral diphenhydramine. Among the seven adverse events, four of these patients received an intravenous graded challenge with ceftriaxone (7.5% of 53 challenges with this agent). The one patient that developed hives was administered a ceftriaxone graded challenge and had previously reported a penicillin allergic reaction of hives. Two patients were transferred to the ICU prior to administration of the intravenous graded challenge due to staff request for enhanced monitoring; neither had a reaction. All 144 intravenous beta-lactam graded challenges were deemed to be appropriate based on the updated AAAAI/ACAAI practice parameter and institutional guidance. Furthermore, 132 (92%) of the patients had their allergy profile updated in the EHR. The median subsequent duration of the challenged antibiotic class was six days with a majority (92/144) of patients receiving a course of ceftriaxone (37%) or cefepime (27%).

**Table 2:** Graded challenge outcomes.

Intravenous graded challenge (*N* = 144)^a^	
**Outcome of IV GC**	
Tolerated without adverse event	137 (95%)
Hives	1 (1%)
Anaphylaxis	0 (0%)
Other^f^	6 (4%)
**Rescue medications administered**	
No	138 (96%)
Yes	6 (4%)
Oral diphenhydramine × 1 dose	6 (100%)
**ICU care due to IV GC**	
No	142 (99%)
Yes^b^	2 (1%)
**Appropriateness** ^e^	144 (100%)
**Updated allergy list in EHR** ^d^	132 (92%)
**Duration of challenged antibiotic class (days), median (range)**	6 (1–98)
**Antibiotic subsequently utilized after IV GC**	
Ampicillin	5 (3%)
Ampicillin/sulbactam	3 (2%)
Aztreonam	3 (2%)
Cefazolin	9 (6%)
Cefdinir	1 (1%)
Cefepime	39 (27%)
Ceftazidime/avibactam	2 (1%)
Ceftolazone/tazobactam	1 (1%)
Ceftriaxone	53 (37%)
Daptomycin	1 (1%)
Ertapenem	7 (5%)
Levofloxacin	3 (2%)
Meropenem	3 (2%)
Nafcillin	1 (1%)
Oxacillin	2 (1%)
Penicillin G	5 (3%)
Piperacillin/tazobactam	6 (4%)

Oral amoxicillin challenge (*N* = 13)^a^	
**Outcome of Oral GC**	
Tolerated without adverse event	13 (100%)
**Rescue medications administered**	
No	13 (100%)
**ICU care due to Oral GC**	
No	13 (100%)
**Appropriateness** ^c^	12 (92%)
**Updated allergy list in EH**R^d^	12 (92%)
**Duration of challenged antibiotic class (days), median (range)**	14 (1–182)
**Antibiotic subsequently utilized after PO GC**	
Amoxicillin	3 (23%)
Amoxicillin/clavulanate	3 (23%)
Ampicillin/sulbactam	1 (8%)
Meropenem	1 (8%)
Penicillin G	3 (23%)
Piperacillin/tazobactam	2 (15%)

^a^ All values are reported as number (percent) unless otherwise noted.

^b^ Both patients were transferred to the ICU prior to administration of the IV GC due to nursing staffing and monitoring requirements.

^c^ One patient was not considered low-risk due to documented PCN allergic reaction as anaphylaxis with no charted date of reaction.

^d^ Allergy either removed or updated to document tolerance/outcome of graded challenge.

^e^ Appropriateness defined as being in alignment with recommendations within the AAAAI/ACAAI updated practice parameter.

^f^ Other reactions included: nausea (3), itching with infusion (2), and sweating (1).

**Table 3:** Subpopulation of patients with reaction to intravenous graded challenge.

Reaction to IV GC (*n* = 7)*	
**IV GC antibiotic administered**	
Ampicillin	1 (14%)
Cefazolin	1 (14%)
Cefepime	1 (14%)
Ceftriaxone	4 (57%)
**Patient reported reaction to penicillin prior to IV GC reaction**	
Rash	2 (29%)
Unknown	2 (29%)
Anaphylaxis	1 (14%)
Hives	1 (14%)
Other	1 (14%)

* All values are reported as number (percent) unless otherwise noted.

Of the 13 oral amoxicillin challenges, all were tolerated without any documented adverse event. No patients required ICU level of care or rescue medications (Table 2). Twelve (92%) of the oral amoxicillin challenges were deemed to be appropriate based on preset criteria (i.e., low-risk patients). One patient was not classified as low-risk due to their documented penicillin allergic reaction as anaphylaxis with no charted date of the reaction; however, the infectious diseases service was consulted and had documented explanation of the oral amoxicillin challenge and patient consent. Twelve (92%) patients had their allergy profile updated in the EHR and the median subsequent duration of the challenged antibiotic class was 14 days. The most common indications for subsequent antibiotic use after administering oral amoxicillin challenges were intra-abdominal abscess (31%) and neurosyphilis (23%).

### Penicillin allergy guidance document & graded challenge order set updates

**Figure 1:**
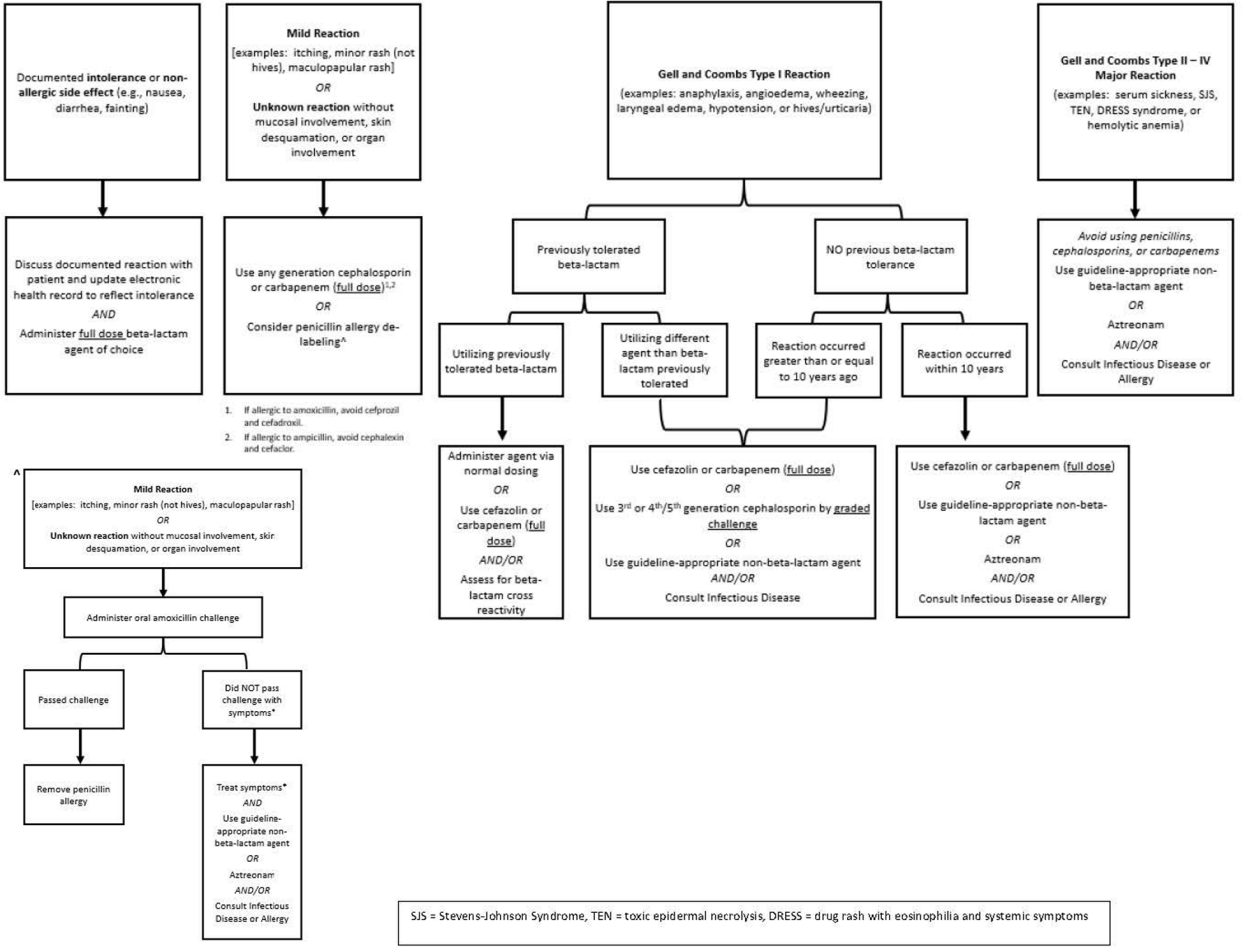
Updated ASP recommendations for challenging penicillin allergic patients.

After the evaluation of graded challenge outcomes, the order set was updated to include oral amoxicillin. Previous institutional guidance did not include recommendations for de-labeling penicillin allergies in the inpatient setting. Therefore our Penicillin Allergy Guidance Document was also revised (Figure 1) to incorporate many of the updates included in the AAAAI/ACAAI Practice Parameter (e.g., emphasis on de-labeling, direct use of cefazolin and carbapenems). Preset orders for cefazolin and ceftazidime/avibactam were added to facilitate future ordering. Future guidance updates will incorporate additional recommendations for patients with allergies to cephalosporins and carbapenems.

## Discussion

We reviewed our institution's intravenous and oral graded challenges and found that in patients with self-reported penicillin or beta-lactam allergies, graded challenges can be safely administered in a hospital setting and were well tolerated in low-risk patients. This aligned with previous literature assessing outcomes from inpatient beta-lactam allergy guidance at multiple institutions that employed intravenous graded challenges, where 5–10% of patients experienced signs or symptoms of an adverse drug reaction and 3%–5% had confirmed hypersensitivity reactions (22–25). At the time of implementation, there was less literature assessing oral amoxicillin challenges in the hospital setting, as most were done on an outpatient basis to assist with penicillin allergy de-labeling (8–21). Thus, this review is unique in its aim to assess the safety and tolerability of both intravenous beta-lactam and oral amoxicillin challenges in a hospital setting.

Most ASP-led beta-lactam allergy interventions in the literature focus on penicillin-skin testing; however, in the absence of this option, graded challenges represent an alternative stewardship opportunity to address beta-lactam allergies. The AAAAI/ACAAI updated drug allergy practice parameter places a greater emphasis on single to multi-step graded challenges and penicillin allergy de-labeling instead of penicillin skin testing (5). This is beneficial logistically and operationally since penicillin skin testing requires dedicated personnel and time, along with special product acquisition and administration. Additionally, through more aggressive penicillin allergy de-labeling and graded challenges, hospitalized patients with documented allergies can receive optimal antimicrobial therapy at the time of suspected infection. Hospitalized patients with documented beta-lactam allergies are more likely to experience inferior outcomes, treatment failures, adverse events, and healthcare-associated infections (26). By utilizing intravenous and oral graded challenges, providers placed patients on either empiric or directed antimicrobial therapy with a preferred beta-lactam agent. Additionally, from an antimicrobial stewardship perspective, administration of intravenous and oral graded challenges avoided empiric aztreonam utilization. A local medication use evaluation demonstrated that aztreonam provided suboptimal activity for many gram-negative organisms according to our institutional antibiogram. Aztreonam was associated with a significant cost burden with an average annual cost in excess of $250,000 to the institution (28). Furthermore, through the successful use of graded challenges, beta-lactams could often be utilized instead of vancomycin or clindamycin, which are associated with less nephrotoxicity and *Clostridioides difficile* infection, respectively.

This review demonstrated that intravenous and oral graded challenges can be administered in a non-ICU level of care and without infectious diseases or allergy consultation. Previous literature supports the safety of both inpatient and outpatient intravenous and/or oral graded challenges (9, 12, 15, 25). This process was implemented at our institution through provider education and buy-in from nursing staff to understand the safety of graded challenges in patients with self-reported beta-lactam allergies. In addition, education to providers improved their understanding and utilization of our ASP Penicillin Allergy Guidance Document. Increasingly over the review period, more non-ID clinicians ordered a graded challenge for a patient with a self-reported beta-lactam allergy. The ability to streamline and implement ASP guidance regarding graded challenges was due, in large part, to the graded challenge order set. This order set has clearly defined monitoring parameters, antimicrobial doses, and rescue medications to ensure patient safety when administering the graded challenge.

Patient allergy profile updates and documentation of successful graded challenge completion occurred for over 90% of patients. Drug allergy documentation and updated drug allergy profiles are important not only for patient safety but also quality of care (27). Inpatient beta-lactam allergy documentation and interventions require an interdisciplinary approach, where all healthcare providers interacting with the patient should be assessing their allergy history and recording it in the EHR. One of the challenges with allergy documentation is that it can be re-added even after a patient has undergone a successful graded challenge. Thus, the ASP team in conjunction with the University of Nebraska Medical Center's Division of Allergy and Immunology identified the opportunity to create a pharmacy consult. Nurses initiate the consult after communication of monitoring parameters to the graded challenge, and pharmacists document a permanent note in the EHR outlining the outcome of the intravenous or oral graded challenge. We believe through this documentation, beta-lactams will be avoided less often, and it may also prevent unnecessary repeated graded challenges.

Finally, this review of graded challenges demonstrated the applicability of implementation, utilization, and adherence to ASP Penicillin Allergy Guidance at an academic medical center. For our intravenous graded challenges, all were considered appropriate and for the oral amoxicillin graded challenges, 92% were appropriate. This demonstrated that among appropriately and carefully selected patients with self-reported penicillin or other beta-lactam allergies, graded challenges were safe and well-tolerated with very low adverse event rates (1% for intravenous graded challenges, none for oral amoxicillin graded challenges). We attribute these lower than previously reported adverse event rates to the close adherence of our institution to our Penicillin Allergy Guidance algorithms (Figure 1) (3). Furthermore, this review demonstrates the safety of a one-step oral amoxicillin challenge, whereas the literature is currently divided regarding the necessity of a two-step oral amoxicillin challenge (8–21). This review shows the applicability and real-world experience of successfully implementing penicillin allergy guidance algorithms and a graded challenge order set in an inpatient setting without direct allergy/immunology consultation.

There are many strengths to our review, but we recognize that there are also several limitations. First, this was a retrospective, single-center review, limiting generalization. Second, the majority of the graded challenges were intravenous, with few oral amoxicillin challenges. However, we saw this as an opportunity to provide updated penicillin allergy guidance that incorporates an oral amoxicillin challenge and additional education to our clinicians on the safety, efficacy, and utility of oral amoxicillin challenges to help de-label penicillin allergies inpatient. Additionally, while these numbers were low, we recognize that this warrants further study to determine if a two-step oral amoxicillin challenge is required instead of the one-step challenge we utilized. Third, we did not compare our graded challenges with penicillin skin testing for patients with self-reported penicillin allergies; however, this is in line with the more recent drug allergy practice parameter and since no patients had documented follow-up with allergy after hospital discharge. Finally, we did not prospectively follow the patients administered a graded challenge to determine how this impacted selection of any future antimicrobial courses after the first. However, during this review period there were no repeated graded challenges in the same patient, which we attribute to the high percentage of updated allergy profiles.

Thus, our review demonstrated that based on previous and current drug allergy practice parameter updates in combination with ASP allergy guidance, both intravenous and oral graded challenges are safe and well-tolerated in an inpatient population.

## Data Availability

The original contributions presented in the study are included in the article/Supplementary Material, further inquiries can be directed to the corresponding author/s.
